# SKP2 cooperates with N-Ras or AKT to induce liver tumor development in mice

**DOI:** 10.18632/oncotarget.2945

**Published:** 2014-12-10

**Authors:** Salvatore Delogu, Chunmei Wang, Antonio Cigliano, Kirsten Utpatel, Marcella Sini, Thomas Longerich, Nina Waldburger, Kai Breuhahn, Lijie Jiang, Silvia Ribback, Frank Dombrowski, Matthias Evert, Xin Chen, Diego F. Calvisi

**Affiliations:** ^1^ Institut für Pathologie, Universitätsmedizin Greifswald, Greifswald, Germany; ^2^ Department of Bioengineering and Therapeutic Sciences and Liver Center, University of California, San Francisco, CA; ^3^ Institute of Pathology, University Hospital Heidelberg, Heidelberg, Germany

**Keywords:** SKP2, AKT, Ras, Hepatocellular carcinoma

## Abstract

Mounting evidence indicates that S-Phase Kinase-Associated Protein 2 (SKP2) is overexpressed in human hepatocellular carcinoma (HCC). However, the role of SKP2 in hepatocarcinogenesis remains poorly delineated. To elucidate the function(s) of SKP2 in HCC, we stably overexpressed the *SKP2* gene in the mouse liver, either alone or in combination with activated forms of N-Ras (*N-RasV12*), AKT1 (*myr-AKT1*), or β-catenin (*ΔN90-β-catenin*) protooncogenes, via hydrodynamic gene delivery. We found that forced overexpression of *SKP2*, *N-RasV12* or *ΔN90-β-catenin* alone as well as co-expression of *SKP2* and *ΔN90-β-catenin* did not induce liver tumor development. Overexpression of *myr-AKT1* alone led to liver tumor development after long latency. In contrast, co-expression of *SKP2* with *N-RasV12* or *myr-AKT1* resulted in early development of multiple hepatocellular tumors in all SKP2/N-RasV12 and SKP2/myr-AKT1 mice. At the molecular level, preneoplastic and neoplastic liver lesions from SKP2/N-RasV12 and SKP2/myr-AKT1 mice exhibited a strong induction of AKT/mTOR and Ras/MAPK pathways. Noticeably, the tumor suppressor proteins whose levels have been shown to be downregulated by SKP2-dependent degradation in various tumor types, including p27, p57, Dusp1, and Rassf1A were not decreased in liver lesions from SKP2/N-RasV12 and SKP2/myr-AKT1 mice. In human HCC specimens, nuclear translocation of SKP2 was associated with activation of the AKT/mTOR and Ras/MAPK pathways, but not with β-catenin mutation or activation. Altogether, the present data indicate that SKP2 cooperates with N-Ras and AKT proto-oncogenes to promote hepatocarcinogenesis *in vivo*.

## INTRODUCTION

Human hepatocellular carcinoma (HCC) is the most frequent primary tumor of the liver and the fifth most common cancer worldwide [1-3]. Due to the late diagnosis and the resistance to conventional chemotherapeutic drugs, the treatment options for unresectable HCC remain limited and the prognosis of the patients is dismal [1-3]. Thus, a better understanding of HCC molecular pathogenesis is mandatory for the identification of novel therapeutic targets and the development of effective treatments against this deadly disease.

Dysregulation of cell cycle-related proteins, leading to unrestrained proliferation, has been shown to be a hallmark of HCC development and progression both in humans and rodents [4-7]. Aberrant regulation of the cell cycle is achieved by liver cancer cells through a program leading to inactivation of tumor suppressor genes responsible for cell cycle arrest and induction of proto-oncogenes promoting cell cycle progression. Indeed, overexpression of cell cycle key genes, including *CYCLINS*, c-Myc, and *E2F1* often coexists with downregulation of cell cycle inhibitors, such as *p16^INK4A^*, *p21^WAF1^*, *p27^KIP1^*, *p53*, *p57^KIP2^*, and *Rassf1A* in liver neoplastic lesions from humans and rodents [4-8]. Such an imbalance leads to pRB hyperphosphorylation and rise in E2F1-DP1 complexes, which activate DNA synthesis genes and allow the transition from the G1 to the S phase of the cell cycle in these lesions [4-8]. Although gene silencing by promoter hypermethylation seems to play a major role in the inactivation of tumor suppressor genes in liver cancer [9-11], emerging evidence indicates a post-transcriptional regulation of cell cycle negative modulators by the S-phase kinase-associated protein 1 (SKP1)/CUL1/F-box protein (SCF) complex, an ubiquitin ligase implicated in the G1-S transition regulation, in this tumor type [12-15]. The pro-oncogenic activity of the SCF complex in various tumors, including HCC, seems to reside in its ability to induce the proteasomal degradation of several inhibitors of the cell cycle, including p27^KIP1^, p57^KIP2^, DUSP1, and RASSF1A [16-21]. In particular, S-phase kinase-associated protein 2 (SKP2), a main member of the SCF complex, is almost ubiquitously overexpressed in cancer and considered to be *a bona fide* oncogene due to its transforming abilities *in vitro* and *in vivo* [16-21]. Previously, others and we have demonstrated that SKP2 is upregulated in rodent and human HCC [12-15]. In addition, it has been shown that SKP2 nuclear accumulation directly correlates with clinical aggressiveness of HCC and is associated with shorter survival of liver cancer patients [15]. In this tumor type, disruption of the negative control operated by kinesin family member 14 (KIF14) on SKP2 seems to be responsible for SKP2 unconstrained activity [22]. Furthermore, *in vitro* data suggest that hepatitis B virus (HBV) core promoter mutations might contribute to HBV associated liver cancer development by SKP2-dependent degradation of the p21^WAF1^ tumor suppressor gene [23]. Altogether, these observations suggest a crucial role of SKP2 in hepatocarcinogenesis. However, the molecular mechanisms underlying SKP2 oncogenic activity remain poorly defined in HCC. In particular, virtually all the functional studies on SKP2 in liver cancer have been performed *in vitro* using HCC cell lines to date. Thus, it remains to be determined whether SKP2 contributes to liver tumor development and/or progression *in vivo*.

Here, we assessed the functional contribution of SKP2 to cancer development *in vivo* by overexpressing *SKP2*, either alone or in association with oncogenes that have been associated with hepatocarcinogenesis, such as activated v-Ras neuroblastoma viral oncogene homolog (*N-Ras)*, v-Akt murine thymoma viral oncogene 1 (*AKT1)*, or *β-catenin*, in the mouse liver via hydrodynamic gene delivery. On the one hand, our results indicate that overexpression of *SKP2* alone is not oncogenic in the mouse liver. On the other hand, we found that SKP2 actively cooperates with N-Ras or AKT, but not with β-catenin, to induce hepatocarcinogenesis in mice by sustaining the activity of the AKT/mTOR and Ras/MAPK pathways.

## RESULTS

### SKP2 cooperates with N-Ras to induce liver tumor development in mice

**Figure 1:**
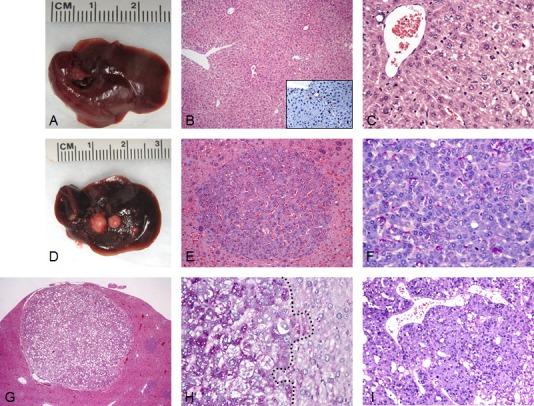
Liver tumor development in SKP2/N-RasV12 mice by hydrodynamic gene delivery (A) Macroscopic and microscopic (B,C) appearance of SKP2-injected livers showing the absence of any gross or histological alterations 40 weeks post hydrodynamic injection. Scattered hepatocytes positive for nuclear HA-tagged SKP2 immunolabeling were present in the liver parenchyma of SKP2 injected mice (B, inset). (D-I) In striking contrast, concomitant overexpression of SKP2 and N-RasV12 resulted in the development of multiple tumors (E-I) in SKP2/N-RasV12 mice. In particular, small tumors (E,F) and large tumors (G-I) were constituted of small cells that were either glycogen-poor (F, as indicated by the PAS staining) or glycogen–rich (H, left part of the picture, demarcated by a dotted line). Tumors were classified as either hepatocellular adenomas (HCA; E,G) or carcinomas (HCC; I). Original magnifications: 40X in G; 100X in B; 200X in inset, E, and I; 400X in C, F, and H.

**Figure 2:**
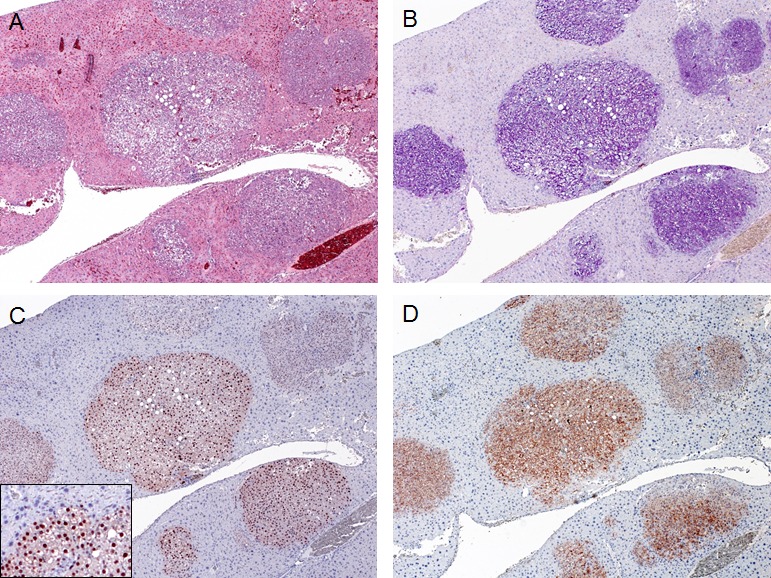
Expression of ectopically injected genes in SKP2/N-RasV12 mice Multiple tumor lesions occurring in a SKP2/N-RasV12 mouse (A; H&E staining) were characterized by glycogen-accumulation (as indicate by PAS staining in B). These tumors were homogeneously immunoreactive for HA-tagged SKP2 (C) and human N-Ras (D), implying their origin from doubly-transfected cells. Note the nuclear staining for HA-SKP2 (C; inset). Original magnification: 100X in A-D; 200X in inset.

To determine whether SKP2 contributes to hepatocarcinogenesis *in vivo*, we hydrodynamically delivered the pT3-EF1α-HA-SKP2 plasmid to the mouse liver either alone or in combination with an oncogenic form of human *N-Ras* (*N-RasV12*). Overexpression of *SKP2* alone did not lead to any tumor formation or histological alteration in mice up to 40 weeks post injection (Fig. 1A-C). At this time point, scattered, single cells positive for HA-tagged SKP2 staining were detected in the liver parenchyma of SKP2 mice (Fig. 1B, inset). Similarly, overexpression of *N-RasV12* alone did not result in any liver anomaly when harvested at the same time point (not shown). In striking contrast, co-expression of *SKP2* and *N-RasV12* genes triggered the development of multiple liver tumors by 20 weeks post injection in all injected mice (Fig. 1D-I). Tumors varied in size and were classified as HCA or HCC based on previously published criteria [24, 25] (Fig.1E-I; Supplementary Table 2). Tumors developed in SKP2/N-RasV12 mice were either composed of basophilic, glycogen-poor cells with small nuclei (Fig. 1E,F) or glycogen-rich cells (Fig. 1G,H). No lesions with cholangiocellular/ductular differentiation were detected in SKP2/N-RasV12 mice. Preneoplastic lesions developed in SKP2/N-RasV12 mice were morphologically equivalent to preneoplastic foci of altered hepatocytes that have been previously described in rat models of chemically-induced hepatocarcinogenesis [26]. The foci developed in these mice were exclusively basophilic, either pure or exhibiting the presence of a small component of clear cell hepatocytes (5-10%) located in the periphery of the focal lesion (Supplementary Fig. 1). Preneoplastic basophilic cells showed cytoplasmic basophilia and were smaller or, more rarely, bigger than normal hepatocytes. The basophilic phenotype was already evident in single hepatocytes or clusters of preneoplastic cells (Supplementary Fig. 1). To confirm that preneoplastic and neoplastic lesions were indeed induced by the ectopically injected oncogenes, we performed immunohistochemistry on SKP2/N-RasV12 lesions by using an anti-HA-tag and an anti-human N-Ras antibody (Fig. 2). As expected, preneoplastic lesions and tumors -but not the normal liver tissue-exhibited strong expression of HA-tag and human N-Ras proteins, implying that tumors originate from the transfected hepatocytes (Fig. 2C,D). Importantly, HA-tag immunoreactivity was localized in the nucleus of transfected cells (Fig. 2C, inset), in accordance with previous findings showing that SKP2 exerts its oncogenic potential at the nuclear level in human HCC [14, 15].

**Figure 3:**
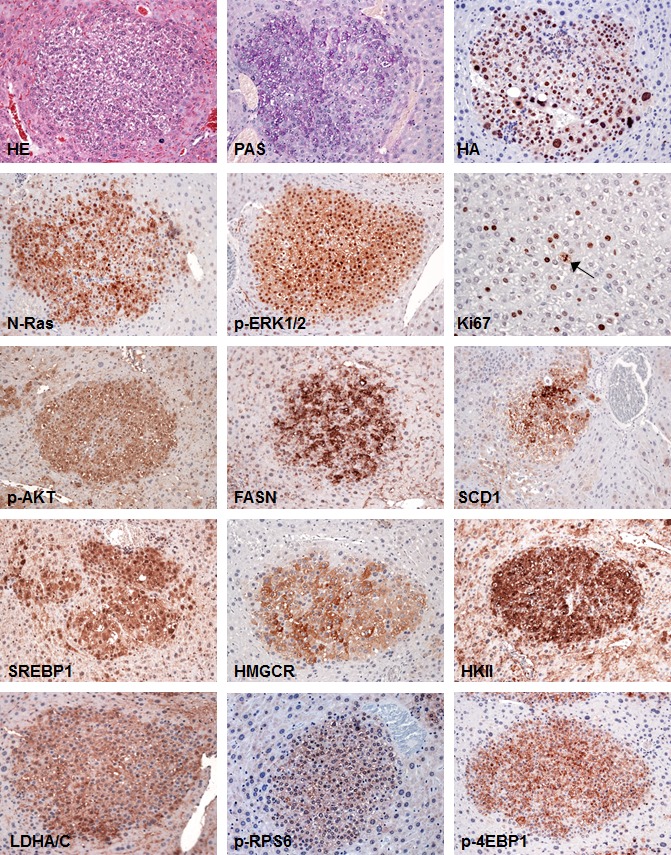
Tumors from SKP2/N-RasV12 mice exhibit strong upregulation of Ras/MAPK and AKT/mTOR pathways SKP2/N-RasV12 preneoplastic and neoplastic lesions showed homogeneous induction of the MAPK pathway, as indicated by strong nuclear accumulation of phosphorylated/activated ERK1/2 (p-ERK1/2) proteins. In addition, these lesions displayed a remarkable activation/phosphorylation of AKT (p-AKT) and its downstream effectors involved in *de novo* fatty acid synthesis (fatty acid synthase or FASN; stearoyl-coA-desaturase 1 or SCD1; sterol regulatory element binding protein 1 or SREBP1), cholesterol synthesis (3-hydroxy-3-methylglutaryl-CoA reductase or HMGCR), glycolysis (hexokinase II or HKII; lactate dehydrogenase A/C or LDHA/C), and protein translation (phosphorylated/activated ribosomal protein S6 or p-RPS6 and phosphorylated/inactivated 4E-binding protein 1 or 4E-BP1). The proliferative activity of these lesions was indicated by positive immunolabeling for Ki67 (the arrow points to a mitotic figure). Serial sections of a small hepatocellular tumor are shown as an example in the present figure. Original magnification: 400X in Ki67-stained liver; 200X in all the other pictures. Abbreviation: HE, hematoxylin and eosin staining.

**Figure 4:**
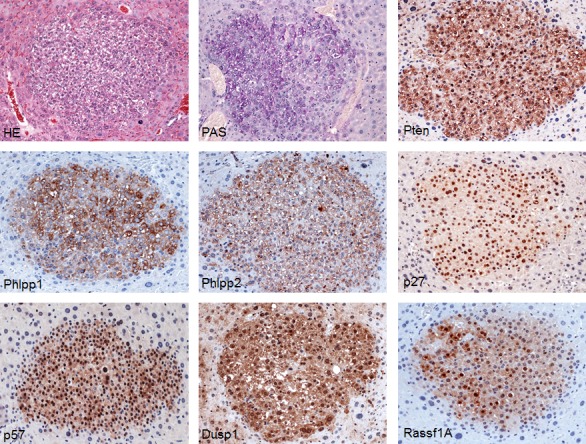
Tumors from SKP2/N-RasV12 mice exhibit strong upregulation of AKT inhibitors (Pten, Phlpp1, and Phlpp2) and putative SKP2 targets (p27, p57, Dusp1, and Rassf1A) Sections of a small hepatocellular tumor already depicted in Fig. 3 are shown as an example in the present figure. Original magnification: 200X in all pictures. Abbreviation: HE, hematoxylin and eosin staining.

To elucidate the molecular mechanisms underlying liver tumor development in SKP2/N-RasV12 mice, we assayed the Ras/MAPK and AKT/mTOR signaling cascades, which have been shown to be prominently activated in human HCC, by immunohistochemistry (Fig. 3). Noticeably, we found that both Ras/MAPK (as indicated by immunoreactivity of activated/phosphorylated ERK1/2) and AKT/mTOR cascades were strongly induced in liver lesions from SKP2/N-RasV12 mice (Fig. 3). In particular, as a consequence of AKT pathway activation, a strong induction of the downstream effectors of this cascade in SKP2/N-RasV12 mice, including proteins involved in glycolysis (hexokinase II, lactate dehydrogenase A/C), *de novo* fatty acid synthesis (fatty acid synthase, stearoyl-coA-desaturase 1, sterol regulatory element binding protein 1), cholesterol synthesis (3-hydroxy-3-methylglutaryl-CoA reductase), and protein translation (phosphorylated/activated ribosomal protein S6 and phosphorylated/inactivated 4E-binding protein 1) was detected, implying an important role of these signaling pathways in SKP2/Ras-driven hepatocarcinogenesis (Fig. 3). Subsequently, since AKT phosphorylation is negatively regulated by the phosphatase and tensin homolog (Pten) as well as the PH domain and leucine rich repeat protein phosphatase 1 and 2 (Phlpp1 and 2) tumor suppressors [27], we determined the levels of these proteins by immunohistochemistry. Interestingly, we found that Pten, Phlpp1, and Phlpp2 were not downregulated, but rather induced, in lesions from SKP2/N-RasV12 mice when compared with the other mouse groups (Fig. 4). These data suggest that activation/phosphorylation of AKT is not a consequence of downregulation of Pten, Phlpp1, and Phlpp2 proteins in SKP2/N-RasV12 mice. Western blot analysis of Ras/MAPK and AKT/mTOR pathways as well as AKT inhibitors revealed a similar pattern to that detected by immunohistochemistry, although the differences were less remarkable presumably due to the “dilution” effect of unaffected liver in protein lysates from SKP2/N-RasV12 mice (Supplementary Fig. 2). Another recognized way whereby SKP2 might induce activation of AKT is through AKT ubiquitination [28]. However, we did not find a significant increase of Akt ubiquitinylation in SKP2/N-RasV12 livers when compared with the other mouse groups (Supplementary Fig. 2), indicating that the latter mechanism is not responsible for increased AKT activity in SKP2/N-RasV12 mice.

Next, we investigated in SKP2/N-RasV12 lesions the levels of tumor suppressors that have been shown to be inactivated in cancer via SKP2-dependent proteolysis, including p27, p57, Dusp1, and Rassf1A [16-21], by immunohistochemistry. Unexpectedly, we found that levels of p27, p57, Dusp1, and Rassf1A were not downregulated -but instead upregulated- in SKP2/N-RasV12 lesions (Fig. 4). Similar, although less remarkable results, were detected by Western blot analysis (Supplementary Fig. 2).

Altogether, the present data indicate that combined overexpression of SKP2 and N-Ras is able to induce tumor development in the mouse liver.

### SKP2 overexpression accelerates AKT-driven hepatocarcinogenesis

**Figure 5:**
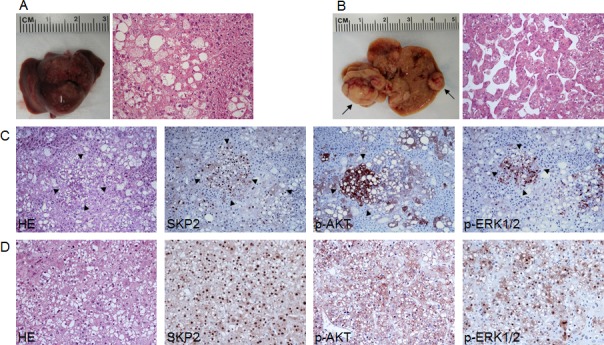
Acceleration of AKT-induced hepatocarcinogenesis in SKP2/myr-AKT1 mice (A) Macroscopic and microscopic appearance of myr-AKT1-injected livers. While macroscopically the liver from these mice appeared normal (left panel), preneoplastic lesions consisting of hepatocytes with enlarged cytoplasm due to intracellular glycogen and lipid accumulation occupied most of the liver parenchyma at this time point (18 weeks post hydrodynamic injection). (B) At the same time point, multiple tumors of various size (indicated by arrows; left panel) were present in the livers of SKP2/myr-AKT1 mice. Most of SKP2/myr-AKT1 tumors were classified as hepatocellular carcinomas (HCC; B, right panel), although few hepatocellular adenomas and cholangiocarcinomas also developed (not shown). (C) Serial sections of a preneoplastic lesion (arrowheads) from a SKP2/myr-AKT1 mouse showing a strong and homogeneous immunoreactivity for SKP2 and phosphorylated/activated AKT (p-AKT) and a sustained activation of the ERK pathway, as indicated by the positive staining for phosphorylated/activated ERK1/2 (p-ERK1/2) proteins. This lesion, consisting of cells with smaller size and less abundant lipid content, is further progressed and closer to neoplastic transformation than the surrounding, large-cell preneoplastic lesions. (D) Serial sections from a solid-type HCC developed in a SKP2/myr-AKT1 mouse exhibiting homogeneous immunolabeling for SKP2, p-AKT, and p-ERK1/2 proteins. Original magnification: 200X in A and B; 100X in C-D. Abbreviation: HE, hematoxylin and eosin staining.

Subsequently, we investigated whether SKP2 synergizes with AKT for tumor development in mice. For this purpose, SKP2 was co-injected with an activated/myristoylated form of AKT1 (myr-AKT1) in the mouse liver via hydrodynamic gene delivery. Only one of six mice injected with the myr-AKT1 plasmid alone developed an HCA by 18 weeks post hydrodynamic injection, whereas all the other myr-AKT1 mice exhibited preneoplastic but not neoplastic lesions (Fig. 5A; Supplementary Table 2). Tumor development was detected in myr-AKT1 mice only 28 weeks after hydrodynamic gene delivery (not shown; Supplementary Table 2), in accordance with previous data [29]. In striking contrast, co-expression of *SKP2* and *myr-AKT1* led to the development of multiple HCC and HCA 18 weeks post injection in all doubly-injected mice (Fig. 5B; Supplementary Table 2). At the histological level, preneoplastic and neoplastic lesions from SKP2/myr-AKT1 mice did not differ significantly from corresponding lesions developed in myr-AKT1 mice. Indeed, preneoplastic hepatocytes showed a pale and enlarged cytoplasm due to lipid accumulation, whereas cell size and lipid content gradually decreased in frankly malignant cells of SKP2/myr-AKT1 mice (Fig. 5C,D). In addition, overexpression of SKP2 did not modify the ratio of tumor types developed in SKP2/myr-AKT1 mice when compared with myr-AKT1 mice. The vast majority of malignant tumors were in fact HCC, whereas cholangiocarcinomas (CCA) rarely occurred in SKP2/myr-AKT1 mice (Supplementary Table 2), in accordance to that described for myr-AKT1 mice [29]. At the molecular level, a strong induction of phosphorylated/activated ERK1/2 proteins was detected in SKP2/myr-AKT1 mice by immunohistochemistry (Fig. 5C,D), whereas myr-AKT1 mouse lesions do not exhibit p-ERK1/2 immunolabeling [30]. Similar to that described in SKP2/N-RasV12 mice, no decline but rather increased levels of putative SKP2 targets, including p27, p57, Dusp1, and Rassf1A was detected in SKP2/myr-AKT1 mice by immunohistochemistry (Supplementary Fig. 3). Similar results were obtained by Western blot analysis (Supplementary Fig. 4). Altogether, the present data indicate that SKP2 accelerates malignant conversion in *AKT* overexpressing livers, presumably by activating the ERK pathway.

### Co-expression of SKP2 and activated β-catenin is not oncogenic in the mouse liver

Next, we determined whether SKP2 cooperates with the Wnt/β-catenin signaling to induce liver tumors in mice. For this reason, we hydrodynamically delivered an oncogenic form of β-catenin (*ΔN90-β-catenin*) with a MYC tag either alone or in combination with *SKP2* in the mouse liver. ΔN90-β-catenin has been previously shown to be unable to induce liver tumors in mice alone, but it promotes HCC development in association with myr-AKT1, c-Met, or N-RasV12 protooncogene [31-33]. Of note, neither overexpression of *ΔN90-β-catenin* alone (not shown), nor co-expression of *ΔN90-β-catenin* and *SKP2* resulted in any histologic alteration in the mouse liver by 28 weeks post-injection (Supplementary Fig. 5; Supplementary Table 2). While immunoreactivity for MYC-tag (ΔN90-β-catenin) was not detectable in livers from ΔN90-β-catenin mice at this time point (not shown), single cells exhibiting MYC-tag (ΔN90-β-catenin) and HA-tag (SKP2) immunolabeling were detected in livers from SKP2/ΔN90-β-catenin mice (Supplementary Fig. 5). These cells did not show any morphological alteration when compared with normal hepatocytes and, therefore, the co-localization of MYC-tag and HA-tag staining was difficult to achieve. Nevertheless, the percentage of cells positive for MYC-Tag and HA-tag was counted and found to be equivalent in SKP2/ΔN90-β-catenin mice (3.0±0.5 and 3.2±0.4, respectively; n=5), implying that the same cells express the two plasmids. Furthermore, the percentage of cells positive for the two constructs is in line with the percentage of hepatocytes targeted by hydrodynamic gene delivery [34]. This observation, together with the absence of proliferation in SKP2/ΔN90-β-catenin livers (not shown), suggests that SKP2/ΔN90-β-catenin cells persist in the mouse liver but do not possess oncogenic properties allowing tumor formation.

### Nuclear localization of SKP2 correlates with activation of AKT/mTOR and Ras/MAPK pathways but not β-catenin cascade in human HCC

**Figure 6:**
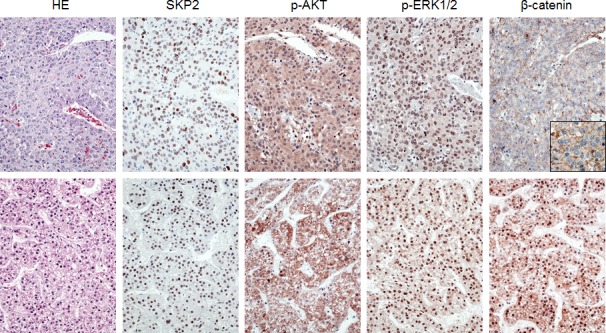
Immunohistochemical patterns of SKP2, activated/phosphorylated AKT (p-AKT), activated/phosphorylated ERK1/2 (p-ERK1/2), and β-catenin proteins in human hepatocellular carcinoma (HCC) Upper panel: HCC specimen showing strong immunolabeling for nuclear SKP2, p-AKT, and p-ERK1/2 and membranous β-catenin (better appreciable in the inset). Lower panel: HCC specimen exhibiting strong immunoreactivity for nuclear SKP2, p-AKT, p-ERK1/2, and nuclear β-catenin. Abbreviation: HE, hematoxylin and eosin staining. Original magnification: 400x in inset; 200x in all the other pictures.

To further investigate the possible relationship between SKP2 and the AKT/mTOR, Ras/MAPK, and Wnt/β-catenin pathways, we analyzed a collection of human HCC specimens (n = 64; Supplementary Table 3) by immunohistochemistry for SKP2, p-AKT, p-ERK, and β-catenin staining (Fig. 6). SKP2 nuclear accumulation was detected in 18 of 64 HCC (28.1%), whereas positive immunolabeling for p-AKT, p-ERK, and nuclear β-catenin was found in 54.7%, 32.8%, and 29.7% of HCC, respectively. β-catenin mutations were detected in 12 of 64 HCCs (18.8%). Of note, 83.3% (15/18) and 66.6% (12/18) of HCCs displaying nuclear accumulation of SKP2 concomitantly showed up-regulation of p-AKT and p-ERK, respectively. In contrast, only 16.7% (3/18) of HCC specimens with nuclear SKP2 concomitantly exhibited nuclear accumulation of β-catenin (P < 0.001 and P < 0.01 vs. p-AKT and p-ERK, respectively). Of the samples showing simultaneous nuclear SKP2 and β-catenin immunoreactivity, two harbored β-catenin mutations. Importantly, 11 of 13 HCCs showing concomitantly nuclear SKP2 accumulation and p-ERK and p-AKT activation belonged to the HCC subset with poorer outcome, suggesting that simultaneous activation of the latter cascades is associated with a dismal prognosis in HCC. No association between the staining patterns of SKP2, p-AKT, and p-ERK and other clinicopathologic features of the patients, including etiology, presence of cirrhosis, α-fetoprotein levels, and tumor grading was found. Subsequent analysis of an additional collection of human HCC (n = 44) whose survival data were missing, showed nuclear accumulation of SKP2 in 11 of 44 (25%) samples, and activation of p-AKT, p-ERK, and β-catenin in 59.1%, 31.9%, and 36,4%, respectively. β-catenin mutations were detected in 11 of 44 HCCs (25%). Once again, nuclear accumulation of SKP2 was frequently paralleled by p-AKT and p-ERK activation, as eight of 11 HCC exhibited concomitantly nuclear SKP2 and p-AKT and p-ERK immunoreactivity, whereas only two HCC displayed concomitant nuclear SKP2 and β-catenin accumulation (P < 0.05). Altogether, the present data indicate that HCC with nuclear SKP2 translocation are often characterized by activation of the AKT/mTOR and Ras/MAPK pathways, with β-catenin mutations and/or activation rarely occurring in this HCC subset.

## DISCUSSION

Emerging evidence supports an important role of the proteasome/ubiquitin machinery in carcinogenesis. A pro-oncogenic function of this system has been suggested by the finding of strong upregulation of members of the proteasome/ubiquitin complex both in experimental and human tumors [19, 20]. In particular, overexpression of SKP2, a prominent member of the SCF complex, is ubiquitous in cancer and often associated with tumor biological aggressiveness [16-18, 21]. The oncogenic capacity of SKP2 has been proven both by *in vitro* and *in vivo* approaches. For instance, it has been shown that SKP2 cooperates with H-RasG12V to trigger both cellular transformation in primary rodent fibroblasts and tumor formation in nude mice [35]. Also, although overexpression of SKP2 is not sufficient to malignantly transform the T-cell compartment in the mouse, combined overexpression of SKP2 with N-Ras leads to rapid development of highly aggressive and lethal T-cell lymphomas in these animals [36]. Moreover, forced upregulation of SKP2 promotes prostate cancer cell growth and tumorigenesis in a xenograft model, and overexpression of SKP2 in the mouse prostate leads to prostate intraepithelial neoplasia (PIN) [37]. Based on this body of evidence, it seems plausible that SKP2 acts as a tumor initiator and/or promoter depending on the tissue type and the model used.

As concerns HCC, reports on the oncogenic role of SKP2 are scanty. Upregulation of SKP2 and its partner, CDC28 protein kinase regulatory subunit 1B (CKS1B), has been shown to be associated with a dismal prognosis and unconstrained tumor proliferation in liver cancer [14, 15]. In addition, forced overexpression or silencing of either SKP2 or CKS1B resulted in either growth acceleration or restraint, respectively, in HCC cells [14, 15]. However, these experimental findings rely exclusively on *in vitro* data. Thus, it remained unclear whether SKP2 is a *bona fide* oncogene in the liver and able to malignantly transform hepatocytes *in vivo*. In the present investigation, we have addressed this important issue for the first time. Our data indicate that overexpression of SKP2 *per se* is not sufficient to induce cellular transformation and tumor development in the liver, at least under our experimental setting. Nonetheless, we showed that SKP2 cooperates with other known oncogenes, such as N-Ras and AKT1, to promote liver tumorigenesis. Thus, SKP2 seems to play predominantly a role of tumor promoter rather than tumor initiator in the liver, in accordance with the data obtained in the T-cell lymphoma murine model [37]. In line with the latter observation, we found that accelerated tumor progression in AKT/Ras mice is associated with increased levels of SKP2 when compared with slowly growing preneoplastic and neoplastic liver lesions from AKT mice [38]. Of note, our findings also demonstrate that SKP2 synergizes with some but not all oncogenes to induce liver tumor development. Indeed, our data indicate that co-expression of SKP2 with β-catenin, a gene frequently activated in mouse and human HCC [39] and whose overexpression cooperates with other oncogenic events to induce HCC development [31-33], does not result in any histological change in the mouse liver. Importantly, similar to that described in mice, concomitant SKP2 nuclear accumulation and β-catenin activation is a rare event in human HCC specimens. In contrast, human (and mouse) liver tumors with SKP2 nuclear translocation frequently exhibited the simultaneous activation of the AKT/mTOR and Ras/MAPK cascades. This body of evidence suggests that the combination of specific molecular events concurs to promote hepatocarcinogenesis, whereas others do not synergize for this scope.

At the molecular level, the oncogenic potential of SKP2 seem to reside in its ability to promote the degradation of the protein products of various tumors suppressor genes involved in the control of cell cycle and apoptosis induction, such as p27, p57, Dusp1, and Rassf1A [16-21]. Importantly, the latter tumor suppressor genes were not downregulated in liver lesions from SKP2/N-RasV12 and SKP2/myr-AKT1 mice. Although p27 downregulation is frequently observed in various types of tumors as a consequence of SKP2 overexpression [16-21], our data are in agreement with recent evidence showing that skin carcinogenesis is inhibited by SKP2 deficiency in a p27-independent manner [40]. Similar to its partner CKS1B, which contributes to carcinogenesis via proteasome-dependent and independent mechanisms [41], SKP2 might exerts its oncogenic potential also via mechanisms independent of its ligase activity. In this regard, it has been recently shown that SKP2 contributes to RhoA transcriptional activation in an E3 ligase-independent manner [42]. Our preliminary data, however, speak against an upregulation of RhoA in SKP2/N-RasV12 and SKP2/myr-AKT1 mice (Delogu S et al., unpublished observation). Another possible explanation to the lack of tumor suppressor proteolysis in SKP2/N-RasV12 and SKP2/myr-AKT1 mice is that overexpression of SKP2 alone is not sufficient to trigger an effective ubiquitinylation program in the mouse liver and, presumably, some other SKP2 partners require to be concomitantly overexpressed to achieve this goal. In agreement with this hypothesis, SKP2 and its partners, CKS1B and SKP1 are strongly induced in HCC [14, 15, 41]. Nonetheless, a pilot study conducted in our laboratory has recently shown that co-expression of SKP2, N-RasV12, and p27T187A -an unphosphorylatable/not degradable form of p27 [43]- in the mouse liver via hydrodynamic gene delivery, neither delayed nor inhibited hepatocarcinogenesis in SKP2/N-RasV12/p27T187A mice (n=3) when compared with SKP2/Ras mice (Calvisi D et al, unpublished observation). The latter finding, which requires additional validation, suggests that SKP2 can act as an oncogene independent of its ability to degrade p27. Among the molecular mechanisms that might play a crucial role in SKP2-associated hepatocarcinogenesis, we found a strong activation of the AKT/mTOR and Ras/MAPK cascades in preneoplastic and neoplastic lesions from SKP2/N-RasV12 and SKP2/myr-AKT1 mice. Others and we have previously shown that SKP2 is able to sustain ERK1/2 activity by suppressing Dusp1, a phosphatase involved in ERK proteins inactivation, *in vitro* [12, 44]. However, the absence of Dusp1 downregulation in liver lesions from SKP2/N-RasV12 and SKP2/myr-AKT1 mice suggest that SKP2 supports ERK activation also via Dusp1-independent mechanisms. In addition, SKP2 overexpression does not lead to upregulation of upstream inducers of the ERK cascade, including EGFR, ERBB2, or c-Met, in SKP2/N-RasV12 and SKP2/myr-AKT1 (Delogu S et al., unpublished observation). Nonetheless, the activation of the ERK pathway is crucial for hastening AKT-induced hepatocarcinogenesis in SKP2/myr-AKT1 mice, as ERK forced induction triggers a tremendous acceleration of liver tumor development and progression in myr-AKT1/Ras mice when compared with myr-AKT1 mice [38]. As concerns AKT activity, the latter can be negatively regulated by a number of phosphatases, such as Pten, Phlpp1, and Phlpp2 [27]. However, our data indicate that activation of AKT is associated with upregulation rather than downregulation of Pten, Phlpp1, and Phlpp2 proteins in SKP2/N-RasV12 mice. This observation suggests the existence of alternative mechanisms responsible for AKT activation that might override the compensatory induction of Pten, Phlpp1, and Phlpp2 proteins in SKP2/N-RasV12 mice. Similarly, we have no evidence that AKT activation is driven by SKP2-dependent ubiquitination in SKP2/N-RasV12 mice. Thus, the precise molecular mechanisms whereby SKP2 activates the AKT/mTOR and Ras/MAPK pathways require additional investigations.

In summary, we showed for the first time that SKP2 cooperates with N-Ras and AKT oncogenes to promote hepatocarcinogenesis in the mouse. In SKP2/N-RasV12 and SKP2/myr-AKT1 mice, the molecular substrates of SKP2 in mediating its oncogenic activity presumably differ from the canonical targets of SKP2 previously identified in many tumor types. Proteomic approaches might be helpful for the identification of SKP2 targets specifically required for SKP2-mediated tumorigenesis. Together with previous data obtained in HCC cell lines and human HCC [12, 14], the present findings substantiate the hypothesis that SKP2 represents a promising therapeutic target in this deadly disease. Noticeably, recently developed soluble inhibitors of SKP2 have shown encouraging anti-neoplastic effects in various tumor types [45, 46]. Thus, it is conceivable that novel therapeutic strategies combining SKP2 inhibitors with AKT/mTOR and Ras/MAPK inhibitors might be highly beneficial for the treatment of human liver cancer.

## MATERIALS AND METHODS

### Constructs

Most of the constructs used for mouse injection, including pT2-Caggs-NRasV12, pT3-EF1a-myr-AKT1, pT3-EF1a-ΔN90-β-catenin, and pCMV/sleeping beauty transposase (SB) were previously described [31, 38]. HA-tagged human SKP2 was cloned into the pT3-EF1α plasmid via the Gateway polymerase chain reaction (PCR) cloning strategy (Invitrogen, Carlsbad, CA). All plasmids were purified using the Endotoxin free Maxi Prep Kit (Sigma, St. Louis, MO) before injecting into mice.

### Hydrodynamic injection and mouse monitoring

Wild-type FVB/N mice were purchased from the Charles River Laboratory (Wilmington, MA). Hydrodynamic gene delivery was performed as described previously [31, 34]. Briefly, 10 μg of the plasmids encoding the gene(s) of interest along with sleeping beauty transposase in a ratio of 25:1 were diluted in 2 ml saline (0.9% NaCl) for each mouse. Saline solution was filtered through a 0.22 μm filter and injected into the lateral tail vein of 6 to 8-week-old mice in 5–7 seconds. Mice were housed, fed, and monitored in accordance with protocols approved by the Committee for Animal Research at the University of California, San Francisco.

### Histology and immunohistochemistry

Livers were fixed in 4% paraformaldehyde and embedded in paraffin. Preneoplastic and neoplastic liver lesions were assessed by two board-certified pathologists and liver experts (M.E. and F.D.) in accordance with previously established criteria [24, 25]. In contrast to hepatocellular tumors that were usually already visible macroscopically as white nodules, preneoplastic lesions showed no expansive growth. Liver tumor lesions were diagnosed as hepatocellular adenomas (HCAs) if: (1.) the acinar morphology was lost; (2.) the lesion showed a trabecular pattern; (3.) the lesions displayed no or only mild atypia; (4.) the lesions compressed the adjacent parenchyma. Hepatocellular carcinomas (HCCs) were instead diagnosed if the lesion showed either frank signs of malignancy such as mitotic activity, necrosis, widely invasive growth or otherwise a pseudoglandular or a trabecular pattern with more than three cell layers in at least two different areas. In order to determine the type of preneoplastic lesions developed in the mouse models, at least 30 lesions per mouse were counted and evaluated histologically. For immunohistochemistry, deparaffinized sections were incubated in 3% H_2_O_2_ dissolved in 1X phosphate-buffered saline (PBS) for 30 minutes to quench the endogenous peroxidase. For antigen retrieval, slides were microwaved in 10 mM citrate buffer (pH 6.0) for 10 minutes. Subsequently, slides were incubated with primary antibodies (Supplementary Table 1) overnight at 4°C. Only primary antibodies that were previously validated for immunohistochemistry by the manufacturers and highly cited in the literature were applied. The specificity of primary antibody reactivity in immunohistochemistry was further confirmed by either omitting the primary antibody in the immunohistochemical procedure or, when available, by incubating the primary antibody with its specific blocking peptide in a 1:2 dilution for 2 hours at room temperature before adding the primary antibody to the slides. The immunoreactivity was visualized with the Vectastain Elite ABC kit (Vector Laboratories, Burlingame, CA), using Vector NovaRED™ (Vector Laboratories) as the chromogen. Slides were counterstained with Mayer's hematoxylin.

### Western blotting, assessment of ubiquitinylated AKT

Mouse livers were homogenized in lysis buffer [30 mM Tris (pH 7.5), 150 mM NaCl, 1% NP-40,0.5% Na deoxycholate, 0.1% SDS, 10% glycerol, and 2mM EDTA] containing the Complete Protease Inhibitor Cocktail (Roche Molecular Biochemicals, Indianapolis, IN). Protein concentrations were determined with the Bio-Rad Protein Assay Kit (Bio-Rad, Hercules, CA) using bovine serum albumin as standard. For Western blotting, aliquots of 80 μg were denatured by boiling in Tris-Glycine SDS Sample Buffer (Invitrogen, Carlsbad, CA), separated by SDS-PAGE, and transferred onto nitrocellulose membranes (Invitrogen) by electroblotting. Membranes were blocked in Pierce Protein-free Tween 20 Blocking Buffer (ThermoFisher Scientific, Waltham, MA) for 1 h and probed with specific antibodies (Supplementary Table 1). Each primary antibody was followed by incubation with horseradish peroxidase-secondary antibody diluted 1:5000 for 1 h and then revealed with the Super Signal West Pico (Pierce Chemical Co., New York, NY). Equal loading was assessed by reversible Ponceau Red Staining (Sigma-Aldrich, St. Louis, MO) and β-Actin immunoblotting. Additional examples of equal loading are shown in Supplementary Fig. 5. Levels of ubiquitinylated AKT protein were assessed using the UbiQapture®-Q Kit (Enzo Life Sciences, Farmingdale, NY), following the manufacturer's instructions.

### Human liver tissue specimens

A collection of 108 formalin-fixed, paraffin-embedded HCC samples was used in the present study. HCC specimens were kindly provided by Dr Snorri S. Thorgeirsson (National Cancer Institute, Bethesda, MD, USA) and collected at the Institute of Pathology of the University of Greifswald (Greifswald, Germany). Institutional Review Board approval was obtained at the National Institutes of Health and the University of Greifswald. Additional liver tissue specimens were provided by the Tissue Bank of the National Center for Tumor Diseases (NCT, Heidelberg, Germany) in accordance with the regulations of the Tissue Bank and the approval of the Ethics Committee of the University of Heidelberg.

### Assessment of β-catenin mutations and deletions in human HCC specimens

Presence of mutations or deletions in the exon 3 of the human *β-catenin* gene was assessed as described previously [30, 47]. PCR fragments were directly sequenced bi-directionally on an ABI Prism® 377-18 DNA Sequencer (Applied Biosystems, Foster City, CA, USA) using the BigDye® Termination v 1.1 Cycle Sequencing Kit (Applied Biosystems) according to the manufacturer's instructions.

### Statistical Analysis

Student's *t* and Tukey-Kramer tests were used to evaluate statistical significance. Values of P < 0.05 were considered significant.

## SUPPLEMENTARY MATERIAL FIGURES AND TABLES


