# Supplementary material for: SKP2 cooperates with N-Ras or AKT to induce liver tumor development in mice
Source: Oncotarget. 2014 Dec 10;6(4):2222–34. doi: 10.18632/oncotarget.2945 (PMC4385847; doi:10.18632/oncotarget.2945)
Supplement: Supplementary file 1 [file oncotarget-06-2222-s001.pdf]

SKP2 cooperates with N-Ras or AKT to induce liver tumor development in mice

Supplementary Material

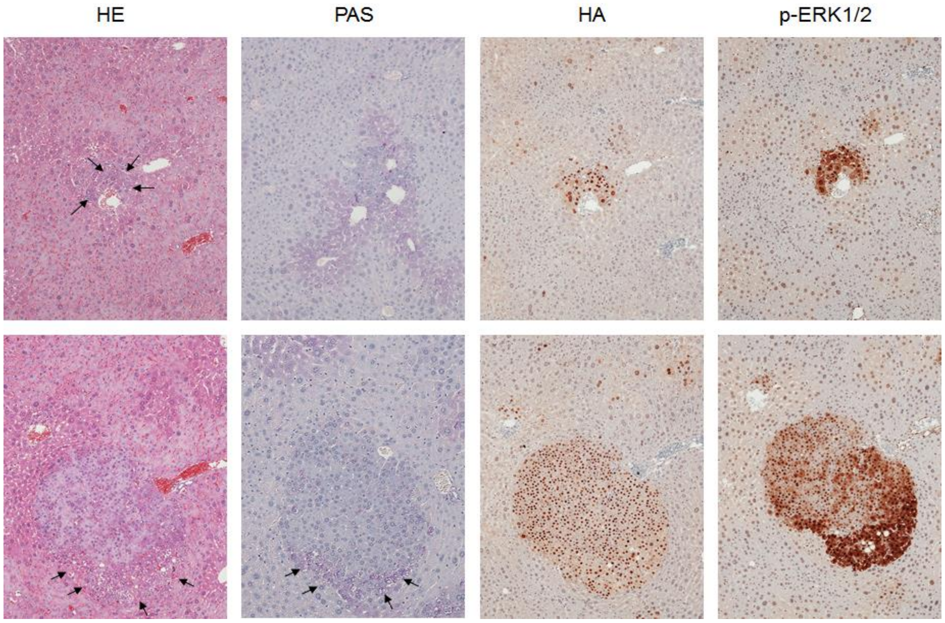

Supplementary Figure 1: Description of preneoplastic lesions occurring in SKP2/N-RasV12 mice. Upper panel: cluster of cells emerging in the pericentral area of a SKP2/N-RasV12-injected mouse liver (indicated by arrows). These cells were characterized by cytoplasmic basophilia and scattered glycogen accumulation (as shown by PAS staining) and expressed the injected plasmids (HA-tag for SKP2, and phosphorylated/activated (p)-ERK1/2 proteins as a surrogate marker of N-Ras activation). Lower panel: SKP2/N-RasV12 mice developed characteristically basophilic foci. In these foci, a small component of clear cell hepatocytes located in the periphery of the focal lesion (indicated by arrows) was frequently detected. These clear cells contained higher amounts of glycogen (indicated by arrows) than the other altered cells (as assessed by PAS staining), and were homogeneously immunoreactive for HA-tag and p-ERK1/2. Original magnification: 200X.

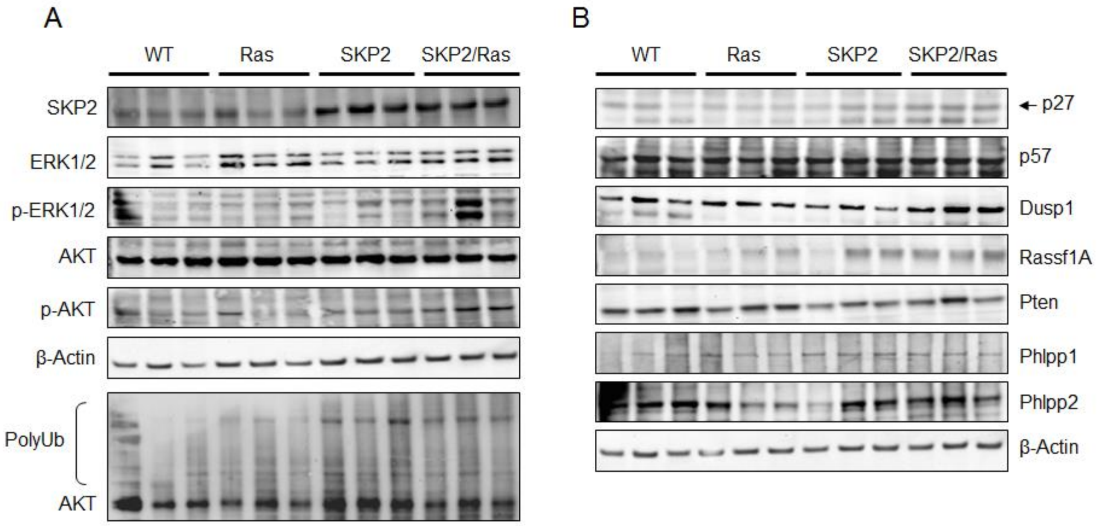

Supplementary Figure 2: Activation of the AKT/mTOR and Ras/MAPK pathways in SKP2/N-RasV12 mice. (A) Western blot analysis of livers from wild-type (WT) mice or injected with SKP2, N-RasV12 or SKP2/N-RasV12. Of note, AKT induction was independent of AKT ubiquitinylation (lower part of A) or PHLPP1, PHLLP2, and PTEN downregulation (lower part of B). Importantly, livers from SKP2/N-Rasv12 mice did not show downregulation of canonical SKP2 targets, including p27, p57, Dusp1, and Rassf1A (B). Four to seven livers from each group of mice were used for the analysis, and representative images are shown. β-Actin was used as loading control. Abbreviations: polyUb, poly-ubiquitinylated.

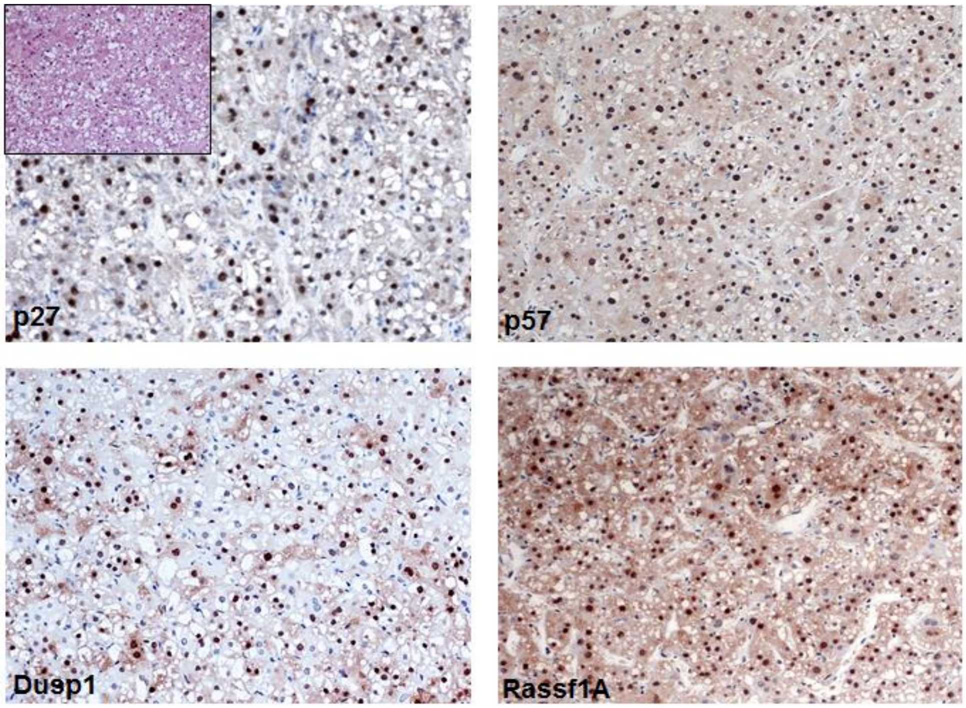

Supplementary Figure 3: Serial sections of the solid-type SKP2/myr-AKT1 HCC from Figure 5 (inset) display a remarkable nuclear accumulation of p27, p57, Dusp1, and Rassf1A tumor suppressors. Original magnification: 100X. Abbreviation: HE, hematoxylin and eosin staining.

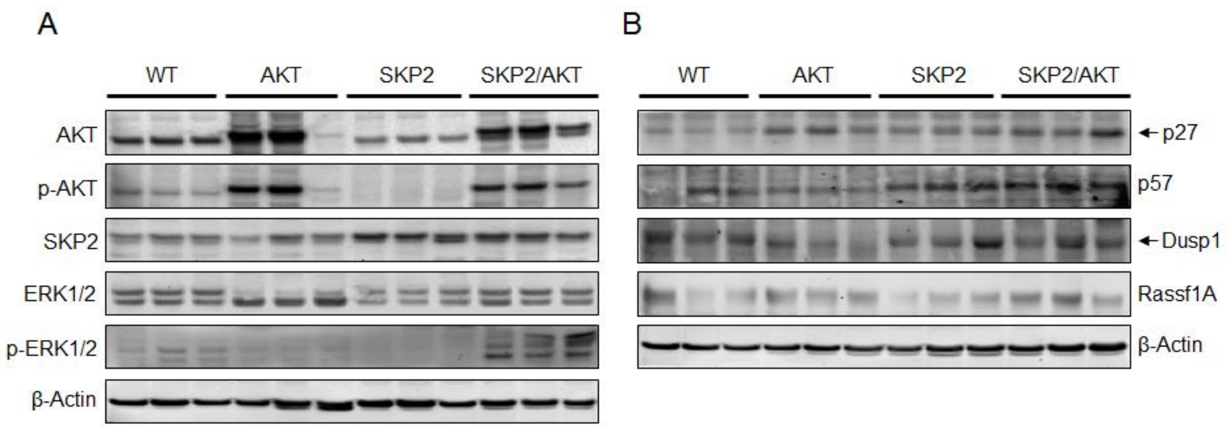

Supplementary Figure 4: Activation of the AKT/mTOR and Ras/MAPK pathways in SKP2/myr-AKT1 mice. (A,B) Western blot analysis of livers from wild-type (WT) mice or injected with SKP2, myr-AKT1 or SKP2/myr-AKT1. As in SKP2/N-RasV12 mice, livers from SKP2/myr-AKT1 mice did not show downregulation of canonical SKP2 targets, including p27, p57, Dusp1, and Rassf1A (B). Four to seven livers from each group of mice were used for the analysis, and representative images are shown. β-Actin was used as loading control.

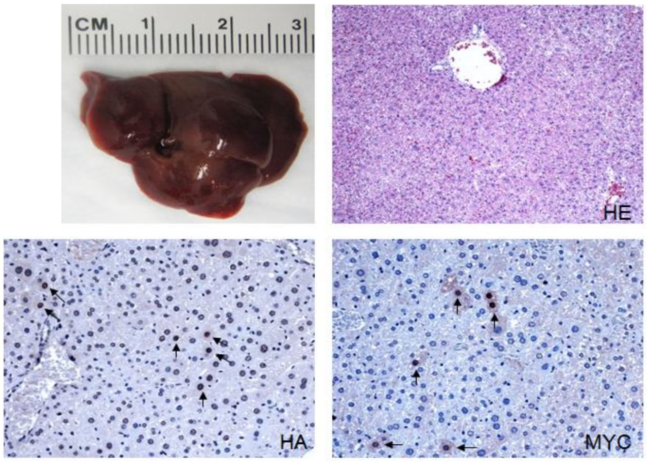

Supplementary Figure 5: Co-expression of SKP2 and oncogenic β-catenin genes in the mouse liver does not result in tumor development. Twenty-eight weeks post hydrodynamic gene delivery, livers from SKP2/ΔN90-β-catenin mice look normal both macroscopically (upper left panel) and microscopically (upper right panel). Lower panels, some hepatocytes (indicated by arrows) express the transfected genes (HA-SKP2 and MYC-ΔN90-β-catenin). Original magnification: 100X for HE; 200X in HA and MYC.

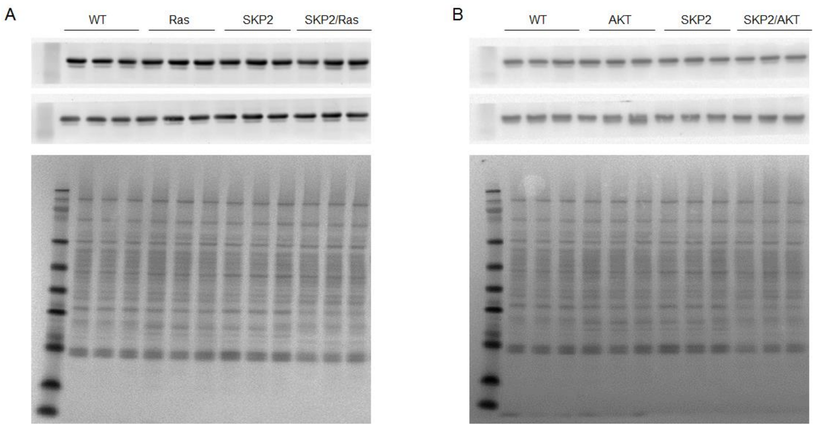

Supplementary Figure 6: Additional, representative examples of equal loading demonstration in Western blot analyses of mouse liver samples (whose identity is indicated above), as assessed by β-actin Western blotting (two upper panels in A and B) and reversible Ponceau Red staining (lower panel in A and B). Abbreviations: WT, wild-type; SKP2/Ras, SKP2/N-RasV12; SKP2/AKT, SKP2/myr-AKT1.

Supplementary Table 1. List of the primary antibodies used for Western blotting (WB) and immunohistochemistry (IHC).

Protein	Antibody (and catalog number)	Epitope mapping	Application
Phospho-AKT	Rabbit monoclonal (13038)	Serine 473[†]	WB; IHC
AKT	Rabbit monoclonal (4691)	COOH terminus[†]	WB
SKP2	Rabbit monoclonal (2652)	NH_2 terminus [†]	WB; IHC
HA-Tag	Mouse monoclonal (2367)	HA-Tag[†]	IHC
N-Ras	Rabbit polyclonal (LS-C99435)	COOH terminus[††]	IHC
Phospho-ERK1/2	Rabbit monoclonal (4370)	pThr202/Tyr204[†]	WB; IHC
SCD1	Rabbit monoclonal (2794)	Full length[†]	IHC
Phospho-4E-BP1	Rabbit monoclonal (2855)	Thr37 and 46[†]	IHC
FASN	Mouse monoclonal (610962)	Amino acids 9-202[*]	IHC
SREBP1	Rabbit polyclonal (sc-8984)	NH_2 terminus[**]	IHC
HMGCR	Mouse monoclonal (sc-271595)	COOH terminus[**]	IHC
HKII	Rabbit monoclonal (2867)	Full length[†]	IHC
LDHA/C	Rabbit monoclonal (3558)	Full length[†]	IHC
p27	Rabbit polyclonal (sc-528)	COOH terminus[**]	IHC; WB
p57	Mouse monoclonal (sc-56341)	Amino acids 45-135[**]	IHC; WB
Dusp1 (MKP-1)	Rabbit polyclonal (sc-1102)	COOH terminus[**]	IHC; WB
Rassf1A	Rabbit polyclonal (ab180801)	Amino acids 1-344[***]	IHC; WB
Pten	Rabbit monoclonal (9188)	COOH terminus[†]	IHC; WB
MYC-Tag	Mouse monoclonal (2276)	MYC-Tag[†]	IHC
β-catenin	Mouse monoclonal (610153)	Amino acids 571-781[*]	IHC
Phlpp1	Rabbit polyclonal (10007191)	Amino acids 1192-1205[†††]	IHC; WB
Phlpp2	Rabbit polyclonal (LS-B6340)	COOH terminus[††]	IHC; WB
Ki67	Rabbit polyclonal (IHC-00375)	Residues 1650-1700[††††]	IHC
β-ACTIN	Mouse monoclonal (A1978)	NH_2 terminus[****]	WB

[†] Provided by Cell Signaling Technology Inc. (Danvers, MA).
[††] Provided by LifeSpan Biosciences (Seattle, WA).
[†††] Provided by Cayman Chemical (Ann Arbor, MI).
[††††] Provided by Bethyl Laboratories, Inc. (Montgomery, TX).
[*] Provided by BD Biosciences (Franklin Lakes, NJ).
[**] Provided by Santa Cruz Biotechnology (Santa Cruz, CA).
[***] Provided by Abcam (Cambridge, MA).
[****] Provided by Sigma-Aldrich (St. Louis, MO).

Supplementary Table 2. Information of the mice used in the present study

Plasmid(s) injected	Harvest time point (post injection)	Preneoplastic lesions	HCA	HCC	CCA
SKP2 (10μg) + SB (0.4μg)	20 weeks	N	N	N	N
SKP2 (10μg) + SB (0.4μg)	20 weeks	N	N	N	N
SKP2 (10μg) + SB (0.4μg)	20 weeks	N	N	N	N
SKP2 (10μg) + SB (0.4μg)	40 weeks	N	N	N	N
SKP2 (10μg) + SB (0.4μg)	40 weeks	N	N	N	N
N-RasV12 (10μg) + SB (0.4μg)	40 weeks	N	N	N	N
N-RasV12 (10μg) + SB (0.4μg)	40 Weeks	N	N	N	N
N-RasV12 (10μg) + SB (0.4μg)	40 Weeks	N	N	N	N
N-RasV12 (10μg) + SB (0.4μg)	40 Weeks	N	N	N	N
SKP2 (10μg) + N-RasV12 (10μg) + SB (0.8μg)	20 weeks	Y	Y (3)	Y (1)	N
SKP2 (10μg) + N-RasV12 (10μg) + SB (0.8μg)	20 weeks	Y	Y (5)	Y (1)	N
SKP2 (10μg) + N-RasV12 (10μg) + SB (0.8μg)	20 weeks	Y	Y (1)	Y (2)	N
SKP2 (10μg) + N-RasV12 (10μg) + SB (0.8μg)	20 weeks	Y	Y (4)	Y (1)	N
SKP2 (10μg) + N-RasV12 (10μg) + SB (0.8μg)	20 weeks	Y	Y (2)	Y (2)	N
SKP2 (10μg) + N-RasV12 (10μg) + SB (0.8μg)	20 weeks	Y	Y (2)	Y (1)	N
SKP2 (10μg) + N-RasV12 (10μg) + SB (0.8μg)	20 weeks	Y	Y (4)	Y (1)	N
SKP2 (10μg) + N-RasV12 (10μg) + SB (0.8μg)	20 weeks	Y	Y (3)	Y (2)	N
Myr-AKT1 (10μg) + SB (0.4μg)	18 Weeks	Y	N	N	N
Myr-AKT1 (10μg) + SB (0.4μg)	18 Weeks	Y	N	N	N
Myr-AKT1 (10μg) + SB (0.4μg)	18 Weeks	Y	N	N	N
Myr-AKT1 (10μg) + SB (0.4μg)	18 Weeks	Y	N	N	N
Myr-AKT1 (10μg) + SB (0.4μg)	18 Weeks	Y	Y (1)	N	N
Myr-AKT1 (10μg) + SB (0.4μg)	18 Weeks	Y	N	N	N
Myr-AKT1 (10μg) + SB (0.4μg)	28 Weeks	Y	Y (1)	Y (3)	N
Myr-AKT1 (10μg) + SB (0.4μg)	28 Weeks	Y	Y (2)	Y (2)	N
Myr-AKT1 (10μg) + SB (0.4μg)	28 Weeks	Y	Y (1)	Y (4)	Y (1)
SKP2 (10μg) + Myr-AKT1 (10μg) + SB (0.8μg)	18 Weeks	Y	Y (1)	Y (2)	N
SKP2 (10μg) + Myr-AKT1 (10μg) + SB (0.8μg)	18 Weeks	Y	Y (2)	Y (1)	N
SKP2 (10μg) + Myr-AKT1 (10μg) + SB (0.8μg)	18 Weeks	Y	N	Y (4)	Y (1)
SKP2 (10μg) + Myr-AKT1 (10μg) + SB (0.8μg)	18 Weeks	Y	Y (1)	Y (4)	N
SKP2 (10μg) + Myr-AKT1 (10μg) + SB (0.8μg)	18 Weeks	Y	Y (1)	Y (3)	Y (1)
SKP2 (10μg) + Myr-AKT1 (10μg) + SB (0.8μg)	18 Weeks	Y	Y (1)	Y (2)	N
SKP2 (10μg) + Myr-AKT1 (10μg) + SB (0.8μg)	18 Weeks	Y	N	Y (4)	N
SKP2 (10μg) + Myr-AKT1 (10μg) + SB (0.8μg)	18 Weeks	Y	Y (1)	Y (4)	N
ΔN90-β-catenin (10μg) + SB (0.4μg)	28 Weeks	N	N	N	N
ΔN90-β-catenin (10μg) + SB (0.4μg)	28 Weeks	N	N	N	N
ΔN90-β-catenin (10μg) + SB (0.4μg)	28 Weeks	N	N	N	N
SKP2 (10μg) + ΔN90-β-catenin (10μg) + SB (0.8μg)	28 Weeks	N	N	N	N
SKP2 (10μg) + ΔN90-β-catenin (10μg) + SB (0.8μg)	28 Weeks	N	N	N	N
SKP2 (10μg) + ΔN90-β-catenin (10μg) + SB (0.8μg)	28 Weeks	N	N	N	N
SKP2 (10μg) + ΔN90-β-catenin (10μg) + SB (0.8μg)	28 Weeks	N	N	N	N
SKP2 (10μg) + ΔN90-β-catenin (10μg) + SB (0.8μg)	28 Weeks	N	N	N	N

Abbreviations: CCA, cholangiocellular carcinoma; HCA, hepatocellular adenoma; HCC, hepatocellular carcinoma; N, No (absence); Y, yes (presence). Number of tumors developed in each mouse is reported in parentheses.

Supplementary Table 3. Clinicopathological features of HCC Patients

Variables	Features	
	HCCB[a]	HCCP[b]
No. of patients	30	34
Male	22	20
Female	8	14
Age (Mean ± SD)	64.2 ± 8.8	68.4 ± 9.2
Etiology		
HBV	15	18
HCV	10	12
Ethanol	5	4
Cirrhosis		
+	24	26
-	6	8
Tumor size		
> 5 cm	22	25
< 5 cm	8	9
Edmondson and Steiner grade		
II	10	9
III	12	14
IV	8	11
Alpha-fetoprotein secretion		
> 300 ng/ml of serum	15	18
< 300 ng/ml of serum	15	16
Survival after partial liver resection (months) Means ± SD	72.0 ± 32.2	22.2 ± 10.2

[a]HCCB, HCC with better outcome/longer survival (survival >3 years following partial liver resection)

[b]HCCP, HCC with poorer outcome/shorter survival (survival <3 years following partial liver resection)
